# A SNP-Based Molecular Barcode for Characterization of Common Wheat

**DOI:** 10.1371/journal.pone.0150947

**Published:** 2016-03-17

**Authors:** LiFeng Gao, JiZeng Jia, XiuYing Kong

**Affiliations:** Key Laboratory of Crop Gene Resources and Germplasm Enhancement, MOA, the National Key Facility for Crop Gene Resources and Genetic Improvement, Institute of Crop Science, CAAS, Beijing, 100081, China; USDA-ARS-SRRC, UNITED STATES

## Abstract

Wheat is grown as a staple crop worldwide. It is important to develop an effective genotyping tool for this cereal grain both to identify germplasm diversity and to protect the rights of breeders. Single-nucleotide polymorphism (SNP) genotyping provides a means for developing a practical, rapid, inexpensive and high-throughput assay. Here, we investigated SNPs as robust markers of genetic variation for typing wheat cultivars. We identified SNPs from an array of 9000 across a collection of 429 well-known wheat cultivars grown in China, of which 43 SNP markers with high minor allele frequency and variations discriminated the selected wheat varieties and their wild ancestors. This SNP-based barcode will allow for the rapid and precise identification of wheat germplasm resources and newly released varieties and will further assist in the wheat breeding program.

## Introduction

DNA fingerprinting is commonly used in crops to identify and characterize cultivars and to protect the rights of breeders [1]. Therefore, several marker systems have been developed for this purpose [2]. RFLPs (restriction fragment length polymorphisms) were the first molecular markers, though this marker system is no longer frequently employed due to the disadvantages of the complexity, low polymorphism rate, and high cost of this technique. SSRs (simple sequence repeats) are considered the second-generation molecular markers. Because they use a comparatively simple technique with a higher polymorphism rate and lower cost, SSR markers have been widely employed in the study of many crops, including wheat [3–5]. The third-generation molecular markers are single-nucleotide polymorphisms (SNPs). With the development of next-generation sequencing (NGS) technology and low-cost genome sequencing, a large number of SNPs are being identified and used to design arrays for major crops [6–11]. SNP arrays are utilized based on a standardized protocol, which makes the resulting data comparable between labs. In theory, SNP arrays comprise loci with unique positions along chromosomes or genomes, thereby largely avoiding the confusion associated with multiple sequence variants. This is especially important for common wheat, a hexaploid plant with three genomes (A, B and D). Recently, several SNP arrays produced by Illumina (9K and 90K) and Affymetrix (35K, 817K, 660K) have been used to evaluate population structure, genetic variation, selection and genome-wide association mapping for agronomic traits in wheat [6, 9, 12, 13]. However, even using medium density SNP arrays to discriminate wheat germplasms or cultivars released every year is still not cost-effective. An economic and easily applied molecular barcode is required for determining cultivar characteristics. Here, we develop a minimal set of SNP markers that is robust enough to fingerprint a diverse collection of wheat genotypes. This SNP-based barcode is composed of 43 SNPs that can resolve 429 wheat accessions, which will facilitate its effective use in wheat germplasm identification and wheat breeding programs.

## Materials and Methods

### Plant materials

Two panels of materials were used in this study. Panel 1 was composed of 429 common wheat cultivars, including land races and modern varieties widely planted across the major wheat-growing areas of China from the 1930s to the 2010s. These cultivars confer important agronomical traits, such as resistance to diseases (stripe rust, sharp eyespot and Fusarium head blight), tolerance to stresses (drought, wet and salt), increased quality, larger grains, longer spikes, and dwarf height (S1 Table). In addition to these remarkable characteristics, some of these cultivars are founder parents from which many modern varieties were bred. The panel 1 samples were used to screen and develop the SNP barcode.

Panel 2 was composed of 193 bread wheat cultivars randomly selected from panel 1 and 96 pairs of wheat ancestor species including wild emmer wheat (*Triticum dicoccoides*) and goat grass (*Aegilops tauschii*) (S2 Table). Panel 2 was used to validate the resolution of the developed SNP barcode.

### DNA extraction and genotyping

DNA was isolated from the leaves of two-week-old seedlings using a DNA extraction kit (CN. DP321, Tiangen Biotech Co., Ltd.). The DNA samples were genotyped using the Illumina wheat 9K Infinium Assay [6]. SNP clustering and genotype calling were performed using GenomeStudio v2011.1 software (Illumina). As described previously [6], the genotyping of polyploid wheat using the 9K SNP chip is complicated by the presence of homologous and paralogous gene copies. Therefore, we manually adjusted the clusters for each SNP using the GenomeStudio software. As suggested [6], when SNP clusters were too close together to allow the AB cluster to be correctly positioned between the AA and BB clusters, one of the HOM (homologous) clusters was defined with the AB cluster; actual HET (heterozygous) genotypes were not called. Then, recoding HOM genotypes was required (e.g., AB to AA). After adjustment of the data as previously described [6], the consistency of genotyping results between labs was largely ensured.

### Genetic analysis of SNP markers

The SNP allele frequency and polymorphism information content (PIC) were estimated for each locus using PowerMarker v3.25 [14]. The PIC value is usually used to estimate the polymorphism for a marker locus among samples. The pairwise locus linkage disequilibrium (LD) was estimated by PowerMarker and Tassel 3.0 [15]. UPGMA trees based on Nei’s genetic distance (GS, Nei’s 1972) were constructed to confirm the resolution capacity of this barcoding system between materials using PowerMarker, and the UPGMA trees were visualized in Mega 5 [16].

### Generation of 2D barcode

We used online software Caoliaoerweima (http://cli.im/) to generate the 2D barcode for each accession. The genotype based on the SNP barcode was entered, and the 2D barcode was automatically generated. Once the barcode was scanned, the genotype of each accession was shown.

### Development of KASP primers

The SNP-based barcode was converted to Kompetitive Allele Specific PCR (KASP) primers, which are specific for SNP genotyping technology (LGC Genomics LLC, Beverly, MA, USA). For each KASP SNP, two allele-specific primers and one common primer were designed (S3 Table). Parameters for primer design were as follows: GC content was less than 60%, melting temperature (Tm) ranged from 55°C to 62°C, and PCR product size was no larger than 120 bp. There were only two choices of allele-specific primers, immediately up- or -down-stream of the SNP site. Therefore, all the primers were manually selected, and GC content and Tm were calculated using DNAstar v7.0. Primers carrying standard FAM- or VIC-compatible tails (FAM tail: 5’-GAAGGTGACCAAGTTCATGCT-3’; VIC tail: 5’-GAAGGTCGGAGTCAACGGATT-3’) with a targeted SNP in the 3’ end were synthesized by Invitrogen Trading (Shanghai). Primer mix was prepared as recommended by Kbioscience: 46 μl ddH_2_O, 30 μl common primer (100 μM), and 12 μl of each tailed primer (100 μM). The total reaction volume was 5 μl in a 384-well plate, composed of 2.43 μl of V3 2× Kaspar mix, 0.07 μl primer mix, and 2.5 μl template (10–20 ng of genomic DNA) as described previously [17]. Ten common wheat varieties were used to test the newly developed KASP primers. PCR was performed as follows: Hot start at 95°C for 15 min, followed by ten touchdown cycles (95°C for 20 s; Touchdown 61°C, -1°C per cycle, 25 s), then followed by 26 cycles of amplification (95°C 10 s; 55°C 60 s). Assays were performed in a QuantStudio 7 Flex Real-Time PCR system, and fluorescence was detected using QuantStudio^TM^ Real-Time PCR software. As suggested by Trick et al. [17], if the signature genotyping groups had not formed after the initial amplification, additional amplification cycles (usually 5–10) were applied, and the samples were read again.

## Results

### SNP marker screening

Of the SNP loci with three typical clusters of AA, AB and BB genotypes, only those loci of the classical bi-allelic type were used for further analysis. According to suggestions on data conversion [6], if raw genotyping data were absent for the AA or BB genotype, AB genotypes were recoded to AA or BB, respectively. After the initial filtering, 3489 SNPs were retained for the wheat accessions in the panel 1.

**Fig 1:**
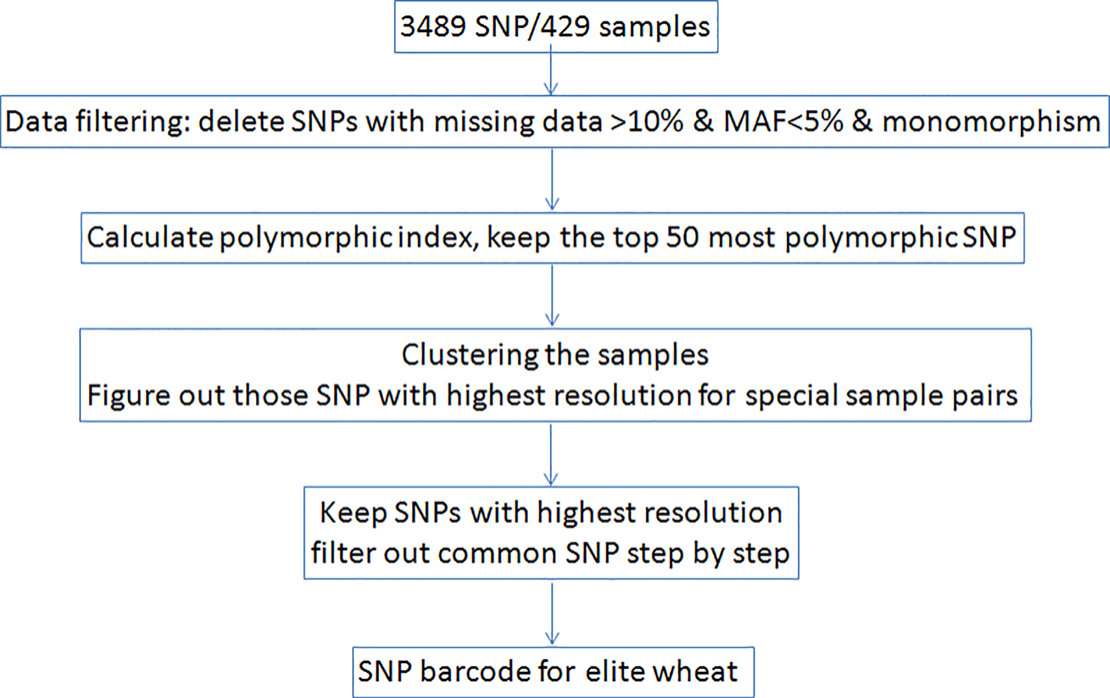
Flow chart of SNP-based molecular barcode screening for bread wheat.

Screening for the SNP barcode was conducted on panel 1 cultivars (Fig 1). First, monomorphic SNPs or those with more than 10% missing data were deleted using PowerMarker software. Then, the PIC value was calculated for each SNP, and the 50 SNP loci with the highest PIC values (hereafter called *original SNP list*) were retained. To evaluate the resolution, genotypes based on the 50 most variable SNP loci were used to construct UPGMA trees for the 429 accessions. Among them, 364 cultivars could be distinguished from each other, accounting for 84.8% of the panel 1 accessions. However, the remaining 15.2% cultivars were not distinguished due to the close relationships among the accessions. For example, Yangmai5 and Funo could not be clearly separated because Funo was a parent of Yangmai5 (Nanda2419/Triumph//Funo///St1472/506) [18]. Further analysis showed that a tiny difference was detected between Yangmai5 and Funo (GS = 0.0003) with 3231 SNP markers from the total 3489 SNPs with no missing data among all the parents of Yangmai5.

Using the UPGMA tree based on the original SNP list, to differentiate the closely related accessions, we identified the SNP loci with different alleles from the other 3439 SNP loci among these accessions. PIC values were calculated for these newly selected SNP markers based on panel 1 accessions. At least one SNP marker with a high PIC value was retained for each pair of closely related accessions. These newly screened SNP loci were sequentially added to replace the SNP markers in the original list. A new UPGMA tree was constructed after each adjustment to the SNP list. In determining the optimal minimum numbers of SNPs used as the SNP barcode, the highest resolution of SNP markers and their distribution across chromosomes were all taken into account. Finally, 43 SNP loci were obtained from the panel 1 accessions to form a SNP-based barcode for hexaploid wheat (Table 1).

**Table 1:** Information about the 43-SNP-based molecular barcode and diversity among panel 1 accessions.

SNP barcode	SNP id^a^	Chr	Position	SNP type	Type_of_ change_I	Type_of_ change_II^b^	429 accessions^c^	
							PIC	MAF
WB01_1A	IWA530	1A	107.3	T/C	Transition	Synonymous	0.35	0.34
WB02_1A	IWA2783	1A	124.5	A/C	Transversion	Nonsynonymous	0.35	0.35
WB03_1A	IWA5405	1A	181.9	T/C	Transition	Synonymous	0.37	0.44
WB04_1B	IWA5186	1B	91.9	A/G	Transition	Synonymous	0.37	0.47
WB05_1B	IWA4153	1B	95.7	T/C	Transition	Nonsynonymous	0.3	0.25
WB06_1B	IWA4154	1B	95.8	T/C	Transition	Nonsynonymous	0.3	0.25
WB07_1B	IWA4155	1B	96.4	T/C	Transition	Synonymous	0.3	0.25
WB08_1B	IWA4031	1B	102.7	A/G	Transition	Nonsynonymous	0.37	0.43
WB09_1B	IWA695	1B	111.2	T/C	Transition	-	0.37	0.48
WB10_1B	IWA4525	1B	133.4	A/G	Transition	-	0.37	0.48
WB11_1D	IWA4884	1D	95.7	T/C	Transition	Synonymous	0.22	0.15
WB12_2A	IWA2938	2A	189.8	A/C	Transversion	Nonsynonymous	0.37	0.49
WB13_2B	IWA2442	2B	47.4	A/C	Transversion	Nonsynonymous	0.36	0.39
WB14_2B	IWA4323	2B	110.8	T/G	Transversion	Synonymous	0.26	0.19
WB15_2D	IWA2961	2D	117.7	A/G	Transition	Synonymous	0.36	0.39
WB16_3A	IWA5285	3A	108.5	T/C	Transition	Synonymous	0.37	0.5
WB17_3B	IWA5106	3B	13.8	A/G	Transition	Synonymous	0.36	0.39
WB18_3B	IWA2037	3B	33.2	T/C	Transition	-	0.26	0.19
WB19_3B	IWA8303	3B	37.2	A/G	Transition	Synonymous	0.34	0.31
WB20_3B	IWA662	3B	52.8	T/C	Transition	-	0.37	0.47
WB21_3B	IWA5618	3B	52.8	T/C	Transition	Synonymous	0.37	0.45
WB22_3B	IWA6279	3B	73.8	A/G	Transition	Synonymous	0.14	0.08
WB23_3B	IWA3710	3B	88.3	A/G	Transition	-	0.37	0.41
WB24_3B	IWA2124	3B	102	A/G	Transition	-	0.37	0.43
WB25_3B	IWA3835	3B	102.6	T/G	Transversion	Nonsynonymous	0.37	0.41
WB26_3D3	IWA3011	3D3	17.7	T/C	Transition	-	0.35	0.34
WB27_4A	IWA1691	4A	108.4	A/G	Transition	Nonsynonymous	0.36	0.37
WB28_4B	IWA4854	4B	41.4	A/C	Transversion	Synonymous	0.34	0.31
WB29_4D	IWA5375	4D	26.9	T/G	Transversion	Nonsynonymous	0.35	0.36
WB30_5A	IWA4805	5A	60.5	T/G	Transversion	Synonymous	0.35	0.34
WB31_5A	IWA5326	5A	123.1	A/G	Transition	Nonsynonymous	0.2	0.13
WB32_5B	IWA1755	5B	96.5	A/G	Transition	Nonsynonymous	0.37	0.49
WB33_5B	IWA2698	5B	111.6	T/C	Transition	Nonsynonymous	0.37	0.46
WB34_5B	IWA6526	5B	158.6	A/G	Transition	Synonymous	0.37	0.44
WB35_5D3	IWA7177	5D3cult	18	T/C	Transition	-	0.37	0.5
WB36_6A	IWA224	6A	114.5	T/C	Transition	-	0.37	0.48
WB37_6B	IWA8380	6B	37.2	A/G	Transition	Synonymous	0.37	0.43
WB38_6B	IWA3312	6B	73.7	T/C	Transition	-	0.37	0.49
WB39_6D3	IWA2476	6D3	6.4	T/G	Transversion	Synonymous	0.35	0.34
WB40_7A	IWA2506	7A	51.2	T/C	Transition	-	0.35	0.35
WB41_7A	IWA4187	7A	145.1	A/G	Transition	Synonymous	0.36	0.39
WB42_7B	IWA1437	7B	19.8	T/C	Transition	Nonsynonymous	0.34	0.32
WB43_7D3	IWA304	7D3	8.5	A/G	Transition	-	0.37	0.5
Mean							0.34	0.37

^a^ SNP id, chromosome position (Chr); SNP type, type of change I and II according to [6, 9]

^b^ -, unknown type of change II

^c^ PIC, polymorphism information content; MAF, minor allele frequency

### Characteristics of the SNP-based molecular barcode

**Fig 2:**
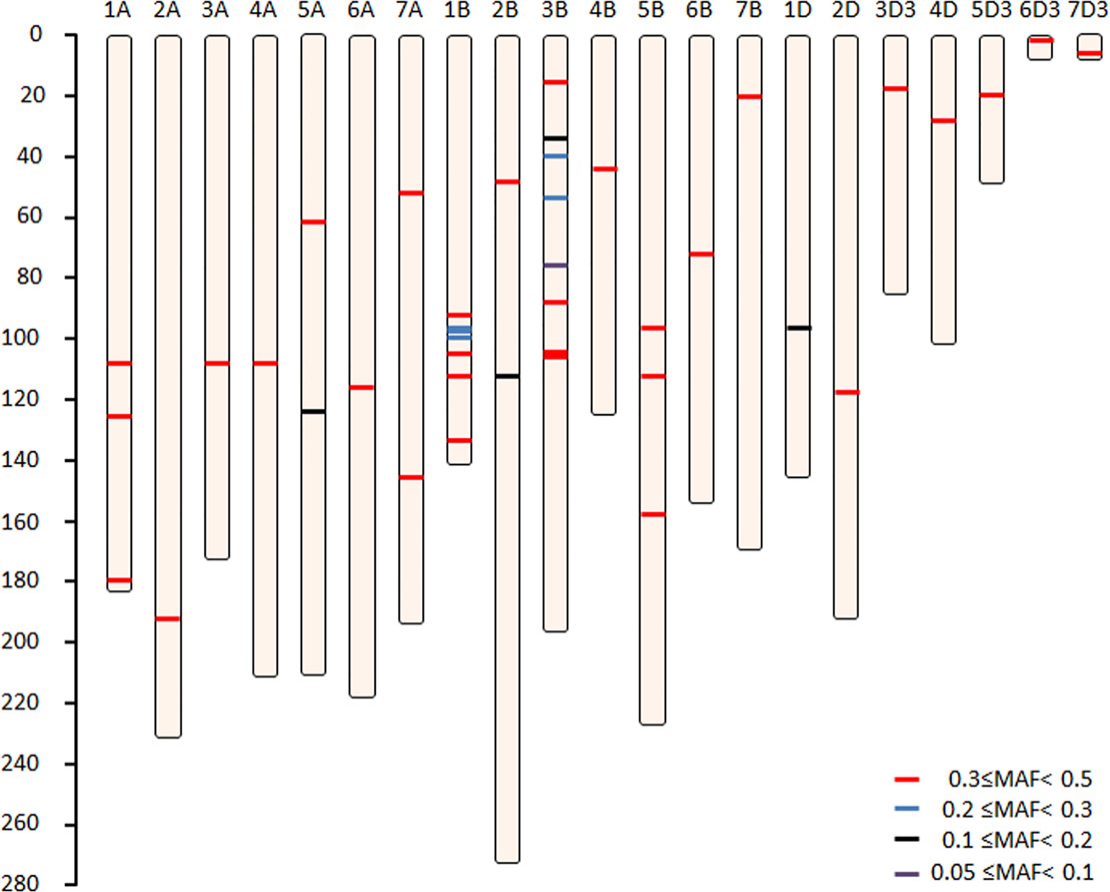
Distribution of the 43-SNP barcode throughout the genome. Each line represents one SNP. The SNP markers are colored according to their minor allele frequency among the panel 1 accessions. The centiMorgan (cM) scale is shown on the left.

**Fig 3:**
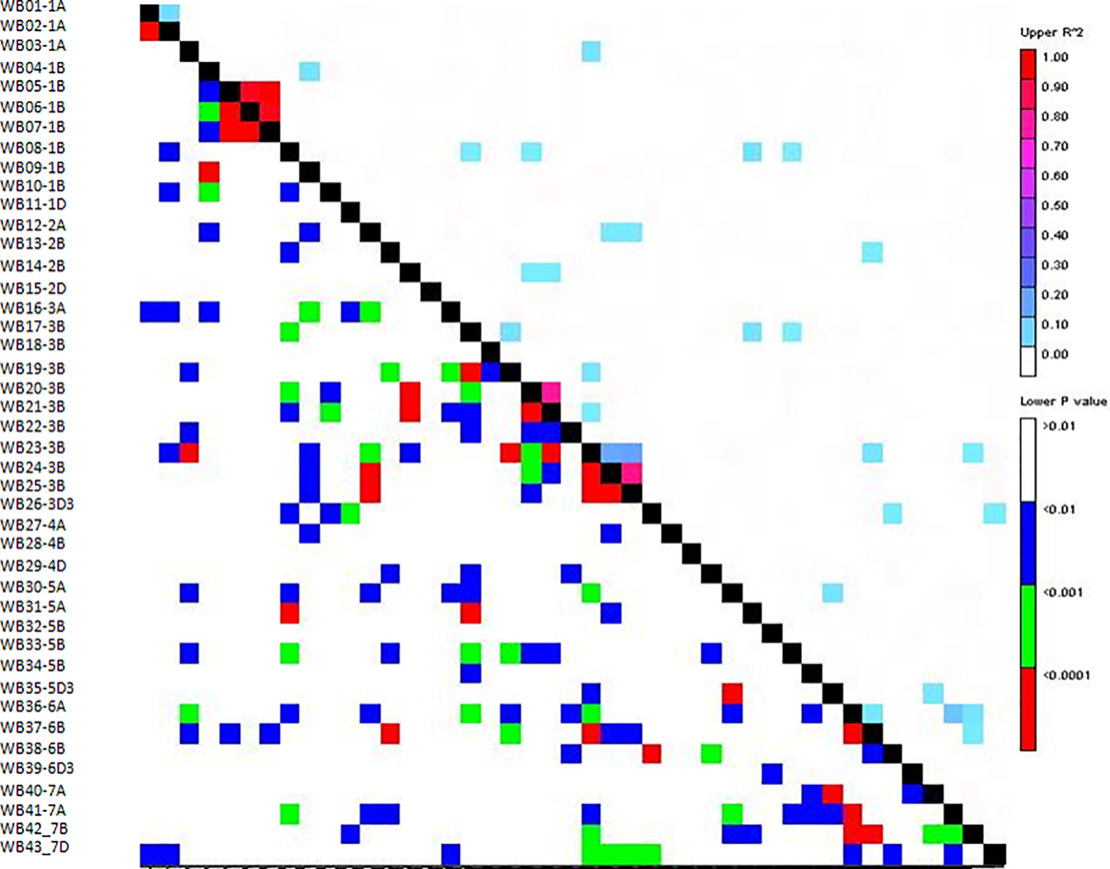
Pair-wise LD for the 43 SNP markers along chromosomes. The SNP information is listed in Table 1.

The 43 SNP loci were disbursed throughout the 21 chromosomes (Fig 2). Relatively, more markers were selected from Chr1B and Chr3B (Table 1) due to the high polymorphism of the B genome between closely related accessions. These SNP loci were generally independent, with loose LD between loci (mean R^2^ = 0.1), though pairs of SNP markers on Chr1B and Chr3B were linked (Fig 3). Of these 43 SNPs, 34 were transitions, 9 were transversions (Table 1), and 18 and 13 SNPs resulted in synonymous and nonsynonymous amino acid changes, respectively. The putative functions of these SNP loci are listed in S4 Table.

The minor allele frequency (MAF) ranged from 8% to 50%, with a mean MAF of approximately 37% across the 429 wheat cultivars. The genetic diversity (PIC) for the 43 SNP loci ranged from 0.14 to 0.38, with a mean PIC value of 0.34. Compared with the original polymorphic data (3489 SNPs), the 43 SNP markers selected showed increased diversity, by approximately 17%, for the panel 1 accessions (PIC values from 0.29 to 0.34) (Table 1). Based on the MAF and PIC values, we could deduce that the 43 SNP markers represent rich variation across wheat cultivars.

### Resolution of the SNP-based molecular barcode

**Fig 4:**
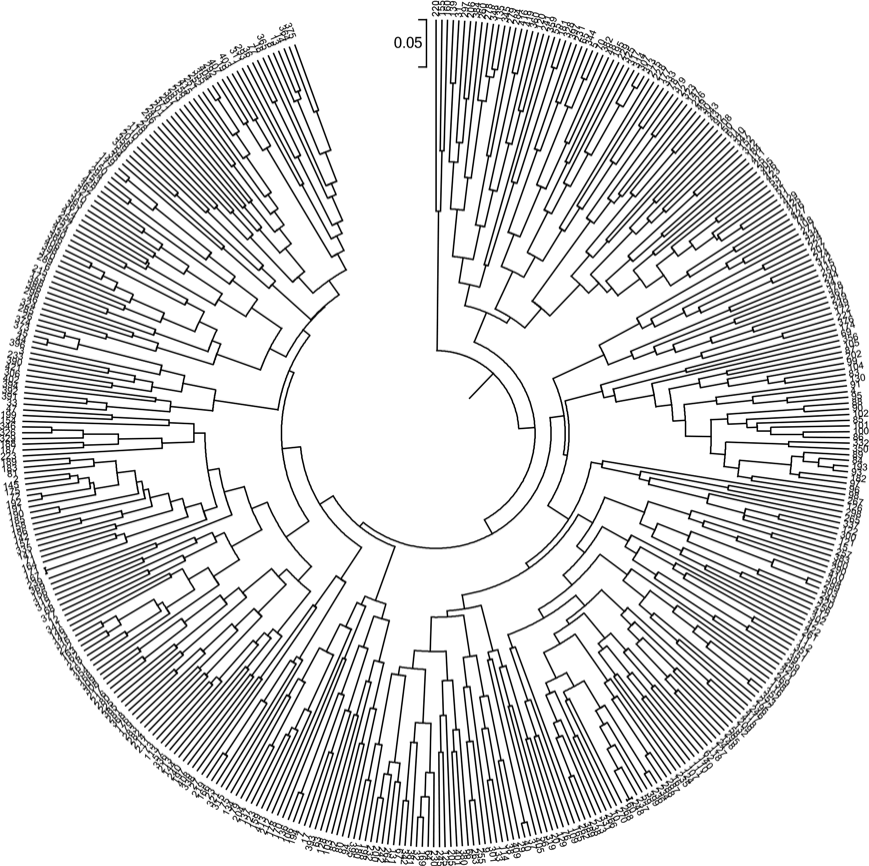
UPGMA trees showed high resolution of the SNP-based barcode. The corresponding accessions for digital number are listed in S1 Table.

**Fig 5:**
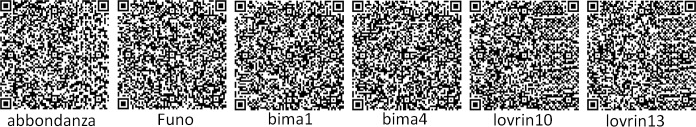
2D barcode for the panel 1 wheat accessions based on 43 SNP markers.

The overall predictive accuracy of the 43-SNP barcode was 100% for the 429 wheat cultivars, distinguishing all the accessions distinctively (Fig 4, S1 Fig). Each accession had its unique and special fingerprint. Among the 429 accessions, the genetic distances ranged from 0.0235 (Mianmai1403 and Mianmai23) to 1.6818 (Nongda311 and Taishan4), and UPGMA trees for the panel 1 accessions indicated that the 43-SNP-based molecular barcode was highly diagnostic. The fingerprints of these accessions demonstrated rich variation and were translated into a 2D barcode that was easily accessible by cell phone (Fig 5).

Usually, DNA barcodes discriminate samples both within and between species. Common wheat is hexaploid (*Triticum aestivum* L.), deriving from spontaneous hybridization of tetraploid *Triticum dicoccoides* (2N = 28, AABB) with diploid *Aegilops tauschii* (2N = 14, DD). The 43-SNP-based barcode was investigated to evaluate its utility for distinguishing accessions with different ploidy. The results indicated that this barcode not only separated common wheat but also distinguished common wheat from its ancestors, as shown by the UPGMA tree (S2 Fig). In addition, the 43 SNP markers discriminated Gansu96, a pedigree of synthetic wheat (durum wheat Lumillo as one parent), from other hexaploid wheat, demonstrating its capability in differentiating genome origin. Unexpectedly, this barcode was sufficiently robust to separate the wheat accession XJ1 from other Chinese landraces. Genetic distance showed that XJ1 was closer to its wheat wild ancestor than to common wheat. XJ1 was collected from the Xinjiang Autonomous Region, which has been suggested as one of the origin sites of common wheat [19]. These results indicated that the high capacity of this 43 SNP-based barcode is not limited to the identification and protection of new varieties.

### Conversion of the SNP markers into KASP probes

KASP technology is highly suitable for the validation of individual SNPs. In this study, we converted 41 of the 43 SNP fingerprinting markers into KASP markers (S3 Table). The remaining two SNPs (WB07 and WB19) could not be converted because the available DNA sequence flanking the SNP was too short or no optimal primer pairs could be designed. Thirty-three of the 41 KASP primers (80%) could be used for the direct genotyping of varieties. Of the 429 common wheat varieties, 425 could be clearly distinguished by the 33 KASP primers (S3 Fig). The two pairs of accessions, Ganmai8 and Longchun8 (coded as 167 and 171, respectively, in S3 Fig) and Han6172 and Heng4399 (coded as 60 and 248, respectively, in S3 Fig), each have a close relationship in that the former is the parent of the latter.

## Discussion

SNPs (single-nucleotide polymorphisms) are the third-generation molecular marker. Their advantages, including high frequency across the whole genome, ease of detection and cost efficiency, make SNP markers particularly popular. With the development of next-generation sequencing (NGS) technology, substantial numbers of SNPs are being discovered, and high diagnostic SNP arrays have been developed for several major crops [6–11]. NGS also provides the opportunity to extend DNA barcoding to new kinds of genomic data [20]. SNP-based barcodes have been developed for human diseases [21–23]. However, to date, no SNP-based barcode has been designed for crops. Here, for the first time, we screened a set of SNPs to develop a SNP-based barcode for wheat. The 43-SNP barcode is rich in variations and has high discriminatory power to discriminate the 429 hexaploid wheat accessions. On average, one SNP can identify ten accessions, a resolution capacity much higher than that reported for ISSRs [24] and SSRs [25], which have usually been used for the identification and evaluation of wheat accessions in the past.

As suggested, diagnostic SNP panels should be composed of the minimal number of SNPs required to differentiate all pairwise comparisons [26]. For SNP arrays with moderate density (i.e., the 90K SNP chip), the cost in consumables for each wheat sample is approximately $100 at current market rates. This is too expensive for breeders who have hundreds of wheat lines to benefit from marker-assisted selection (MAS) based on SNP arrays directly. Therefore, low-density SNP arrays specific for molecular breeding may serve as extended barcodes in the future. In addition to costs, the requirements of such a barcode are that it be stable and easy to operate. In this respect, SNP markers are more easily detectable than SSR markers at present. With the development of the KASP technique (http://www.lgcgroup.com/services/genotyping) specific for detecting single-nucleotide variations, SNP markers can be converted to KASP probes. Thus, SNP detection no longer relies on array technology, and the number of SNP markers depends instead on the requirements of the set of samples for comparison. Using the KASP system, the cost for one SNP per sample is approximately $0.12. In fact, the number of KASP probes or samples per PCR is flexible according to the aims of the experiment. The procedure of PCR amplification is quite simple. Within one to two hours, the genotypes of 384 samples for one SNP marker or one sample for 384 SNP markers will be available. More importantly, KASP probes are labeled by two fluorophores, eliminating the uncertainty of the final data. Any detection systems for Real-time PCR could, in theory, be used for signal capture of KASP probes. Thus, SNP markers detected using the KASP system are less expensive and more efficient than SSR markers [27].

A SNP barcode provides a tool to discriminate very closely related accessions or the origins of wheat subgenomes. In the present study, the wheat accessions used for generating the SNP-based barcode are collections grown in China from the 1930s to the 2010s with important agricultural characteristics. These accessions are rich in variants including landraces and modern cultivars. The 43-SNP-based barcode could discriminate closely related accessions, such as the parent Funo and pedigree Yangmai5. In addition, this SNP barcode could differentiate between genome donors of wheat. For example, the origin of the A and B genomes of Gansu96 is the durum wheat Lumillo, which was detected as disparate from those of other hexaploid wheat cultivars.

By 2006, more than 41,000 wheat accessions had been preserved in the Chinese GeneBank (personal communication). From 2001 to 2015, 340 new varieties were registered according to the national assessment standard, in addition to the varieties released by provinces in China. Therefore, major concerns for wheat researchers and breeders include how to classify the wheat collections in this GeneBank and how to ensure the authenticity and purity of seeds. This 43-SNP barcode may be highly useful in the above work. However, there is still some room to improve this SNP barcode. SNPs of important genes controlling functional divergence as well as SNPs in intergenic regions can be used for designing SNP barcodes in the future.

## Supporting Information

S1 Table Information for panel 1 of 429 wheat accessions: sample code, name, characterization, and genotyping by the 43 SNP markers. (XLSX)

S2 Table Information for panel 2 of 193 common wheat and 96 wild relative pairs: sample code, name, and genotyping by the 43 SNP markers. (XLSX)

S3 Table KASP primers for the SNP-based barcode. (XLSX)

S4 Table Information about the 43-SNP-based barcode: chromosome position and annotation. (XLSX)

S1 Fig Fingerprints of 429 wheat accessions. Each line represents one SNP locus, and each column represents one accession. The SNP and cultivar information is listed in S1 Table. Yellow, green, blue and purple colors represent nucleotides T, A, C and G, respectively. Missing data are indicated by grey color.
(TIF)

S2 Fig UPGMA tree showing that the 43-SNP barcode can distinguish hexaploid wheat from its wild relatives. The black digital numbers are 193 elite cultivars, and the red ones are wild relatives.
(TIF)

S3 Fig UPGMA trees based on 33 SNP markers with working KASP primers. The corresponding accessions for digital numbers are listed in S1 Table.
(TIF)

## References

[1] Wang ZH. DNA fingerprinting technology and its application in crop germplasm resources. Mol Plant Breed. 2006;14: 425–430. (in Chinese)

[2] Grover A, Sharma PC. Development and use of molecular markers: past and present. Crit Rev Biotechnol. 2016;36: 290–302. 10.3109/07388551.2014.959891 25430893

[3] Wu K, Tanksley SD. Abundance, polymorphism and genetic mapping of microsatellites in rice. Mol Gen Genet. 1993;241: 225–235. 7901751 10.1007/BF00280220

[4] Brunel D. A microsatellite marker in *Helianthus annuus* L. Plant Mol Biol. 1994;24: 397–400. 10.1007/BF00020177 8111041

[5] Röder MS, Korzun V, Wendehake K, Plaschke J, Tixier MH, Leroy P, et al. A microsatellite map of wheat. Genetics. 1998;149: 2007–2023. 9691054 10.1093/genetics/149.4.2007 PMC1460256

[6] Cavanagh CR, Chao S, Wang S, Huang BE, Stephen S, Kiani S, et al. Genome-wide comparative diversity uncovers multiple targets of selection for improvement in hexaploid wheat landraces and cultivars. Proc Natl Acad Sci U S A. 2013;110: 8057–8062. 10.1073/pnas.1217133110 23630259 PMC3657823

[7] Chen H, Xie W, He H, Yu H, Chen W, Li J, et al. A high-density SNP genotyping array for rice biology and molecular breeding. Mol Plant. 2014;7: 541–553. 10.1093/mp/sst135 24121292

[8] Unterseer S, Bauer E, Haberer G, Seidel M, Knaak C, Ouzunova M, et al. A powerful tool for genome analysis in maize: development and evaluation of the high density 600 k SNP genotyping array. BMC Genomics. 2014;15: 823 10.1186/1471-2164-15-823 25266061 PMC4192734

[9] Wang S, Wong D, Forrest K, Allen A, Chao S, Huang BE, et al. Characterization of polyploid wheat genomic diversity using a high-density 90,000 single nucleotide polymorphism array. Plant Biotechnol J. 2014;12: 787–796. 10.1111/pbi.12183 24646323 PMC4265271

[10] Hulse-Kemp AM, Lemm J, Plieske J, Ashrafi H, Buyyarapu R, Fang DD, et al. Development of a 63K SNP array for cotton and high-density mapping of intraspecific and interspecific populations of Gossypium spp. G3 (Bethesda). 2015;5: 1187–1209. 10.1534/g3.115.018416 25908569 PMC4478548

[11] Lee YG, Jeong N, Kim JH, Lee K, Kim KH, Pirani A, et al. Development, validation and genetic analysis of a large soybean SNP genotyping array. Plant J. 2015;81: 625–636. 10.1111/tpj.12755 25641104

[12] Faris JD, Zhang Q, Chao S, Zhang Z, Xu SS. Analysis of agronomic and domestication traits in a durum × cultivated emmer wheat population using a high-density single nucleotide polymorphism-based linkage map. Theor Appl Genet. 2014;127: 2333–2348. 10.1007/s00122-014-2380-1 25186168

[13] Zanke CD, Ling J, Plieske J, Kollers S, Ebmeyer E, Korzun V, et al. Whole genome association mapping of plant height in winter wheat (*Triticum aestivum* L.). PLoS One. 2014;9: e113287 10.1371/journal.pone.0113287 25405621 PMC4236181

[14] Liu K, Muse SV. PowerMarker: an integrated analysis environment for genetic marker analysis. Bioinformatics. 2005;21: 2128–2129. 15705655 10.1093/bioinformatics/bti282

[15] Bradbury PJ, Zhang Z, Kroon DE, Casstevens TM, Ramdoss Y, Buckler ES. TASSEL: software for association mapping of complex traits in diverse samples. Bioinformatics. 2007;23: 2633–2635. 17586829 10.1093/bioinformatics/btm308

[16] Tamura K, Peterson D, Peterson N, Stecher G, Nei M, Kumar S. MEGA5: molecular evolutionary genetics analysis using maximum likelihood, evolutionary distance, and maximum parsimony methods. Mol Biol Evol. 2011;28: 2731–2739. 10.1093/molbev/msr121 21546353 PMC3203626

[17] Trick M, Adamski NM, Mugford SG, Jiang CC, Febrer M, Uauy C. Combining SNP discovery from next-generation sequencing data with bulked segregant analysis (BSA) to fine-map genes in polyploid wheat. BMC Plant Biol. 2012;12: 14 10.1186/1471-2229-12-14 22280551 PMC3296661

[18] Chen XH. Yangmai5 and its application. Jiangsu. Agric Sci. 1986 10.1589/j.issn.1002-1302 (in Chinese)

[19] Zhang JB, Wang W, Xiao J, Liu ZY. Study and utilization of wild relatives of wheat in Xinjiang Uyguerautonomous region. Chin Agric Sci Bull. 2011;27: 29–32. (in Chinese)

[20] Coissac E, Hollingsworth PM, Lavergne S, Taberlet P. From barcodes to genomes: extending the concept of DNA barcoding. Mol Ecol. 2016 1 28 Epub ahead of print. 10.1111/mec.13549 26821259

[21] Coll F, McNerney R, Guerra-Assunção JA, Glynn JR, Perdigão J, Viveiros M, et al. A robust SNP barcode for typing *Mycobacterium tuberculosis* complex strains. Nat Commun. 2014;5: 4812 10.1038/ncomms5812 25176035 PMC4166679

[22] Preston MD, Campino S, Assefa SA, Echeverry DF, Ocholla H, Amambua-Ngwa A, et al. A barcode of organellar genome polymorphisms identifies the geographic origin of Plasmodium falciparum strains. Nat Commun. 2014;5: 4052 10.1038/ncomms5052 24923250 PMC4082634

[23] Baniecki ML, Faust AL, Schaffner SF, Park DJ, Galinsky K, Daniels RF, et al. Development of a single nucleotide polymorphism barcode to genotype Plasmodium vivax infections. PLoS Negl Trop Dis. 2015;9: e0003539 10.1371/journal.pntd.0003539 25781890 PMC4362761

[24] Cheng BS, Xu HF, Gu ZZ, Sun SY, Zhou YM, Yang JY. Establishment of SSR fingerprint map and analysis of genetic diversity among main wheat cultivars (*Triticum aestivum* L.) in Huan’an region. Acta Agriculturae Zhengjiangensis. 2011;23: 20–24. (in Chinese)

[25] Hao C, Dong Y, Wang L, You G, Zhang H, Ge H, et al. Genetic diversity and construction of core collection in Chinese wheat genetic resources. Sci Bull. 2008;53: 1518–1526 [in Chinese]. 10.1007/s11434-008-0212-x

[26] Frey JE, Guillén L, Frey B, Samietz J, Rull J, Aluja M. Developing diagnostic SNP panels for the identification of true fruit flies (Diptera: Tephritidae) within the limits of COI-based species delimitation. BMC Evol Biol. 2013;13: 106 10.1186/1471-2148-13-106 23718854 PMC3682933

[27] Hao CY, Wang LF, Jia JZ, Dong YC, Zhang XY. Comparison of fluorescence and silver-staining detection system of microsatellite markers. Acta Agron Sin. 2005;31: 144–149.

