# Supplementary figures and images for: A SNP-Based Molecular Barcode for Characterization of Common Wheat
Source: PLoS One. 2016 Mar 17;11(3):e0150947. doi: 10.1371/journal.pone.0150947 (PMC4795793; doi:10.1371/journal.pone.0150947)

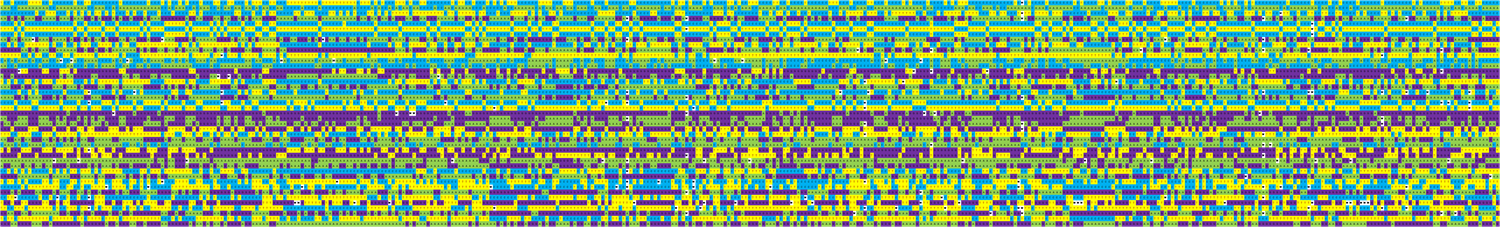

Supplement: S1 Fig — Each line represents one SNP locus, and each column represents one accession. The SNP and cultivar information is listed in S1 Table. Yellow, green, blue and purple colors represent nucleotides T, A, C and G, respectively. Missing data are indicated by grey color. (TIF) [file pone.0150947.s005.tif]

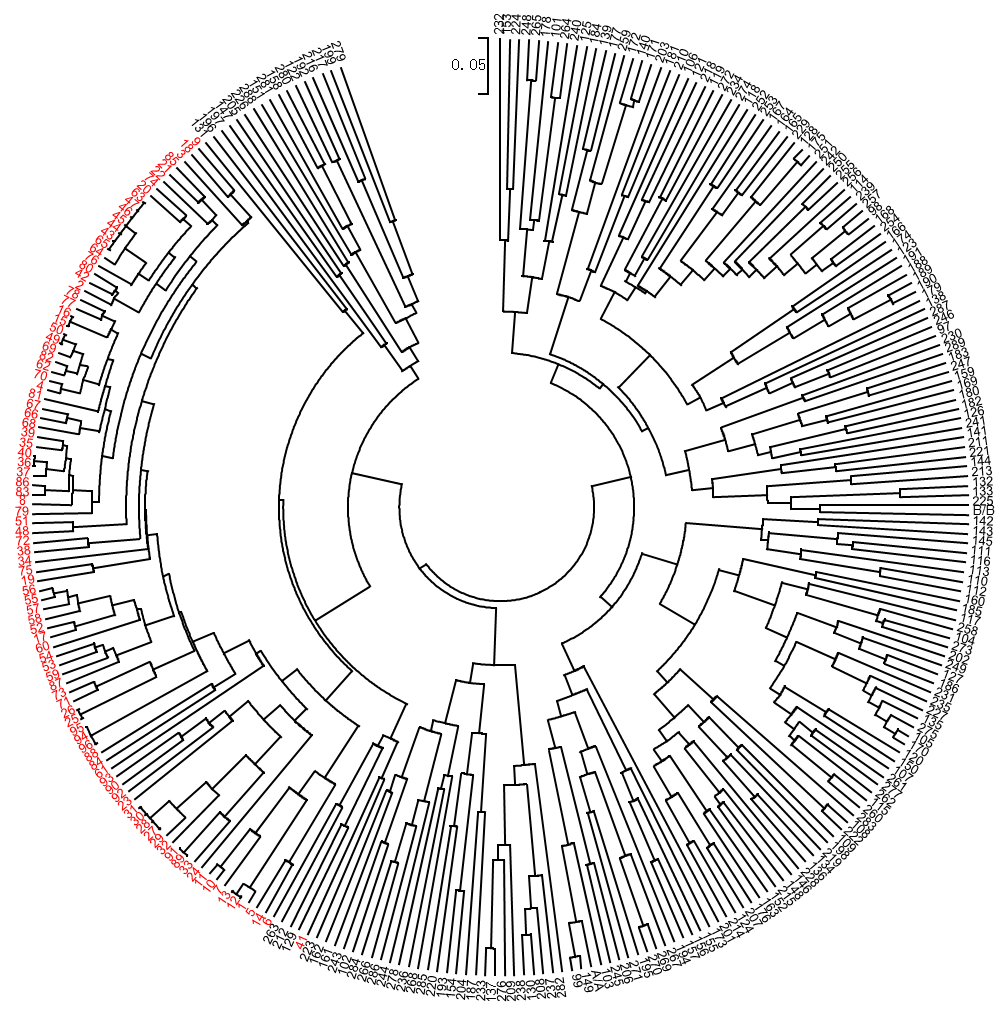

Supplement: S2 Fig — The black digital numbers are 193 elite cultivars, and the red ones are wild relatives. (TIF) [file pone.0150947.s006.tif]

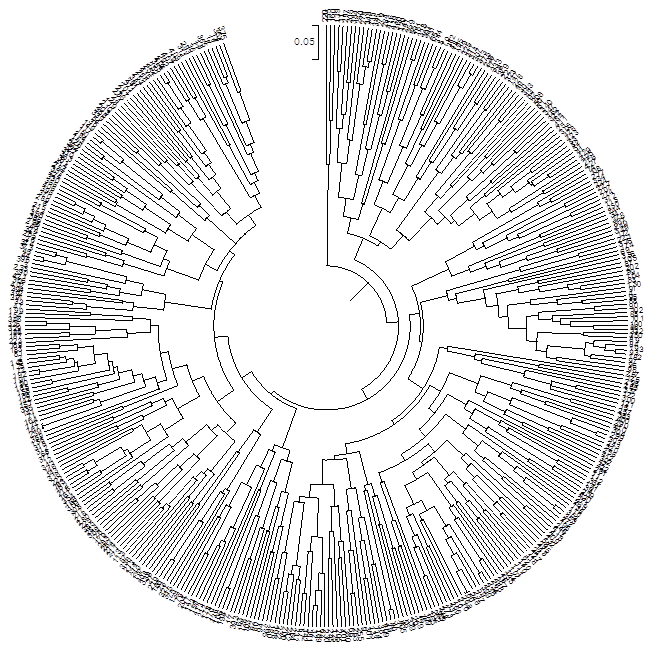

Supplement: S3 Fig — The corresponding accessions for digital numbers are listed in S1 Table. (TIF) [file pone.0150947.s007.tif]
